# Gut microbiota: impacts on gastrointestinal cancer immunotherapy

**DOI:** 10.1080/19490976.2020.1869504

**Published:** 2021-01-12

**Authors:** Harry Cheuk Hay Lau, Joseph Jao-Yiu Sung, Jun Yu

**Affiliations:** Institute of Digestive Disease, Department of Medicine and Therapeutics, State Key Laboratory of Digestive Disease, Li Ka Shing Institute of Health Sciences, CUHK Shenzhen Research Institute, the Chinese University of Hong Kong, Sha Tin, Hong Kong

**Keywords:** Gut microbiota, gastrointestinal cancer, adoptive cell transfer, immune checkpoint blockade, blockade-induced adverse events, CpG-oligodeoxynucleotide therapy, probiotics, fecal microbiota transplantation

## Abstract

The association of gut microbiota with gastrointestinal carcinogenesis has been heavily investigated since the recent advance in sequencing technology. Accumulating evidence has revealed the critical roles of commensal microbes in cancer progression. Given by its importance, emerging studies have focussed on targeting microbiota to ameliorate therapeutic effectiveness. It is now clear that the microbial community is closely related to the efficacy of chemotherapy, while the correlation of microbiota with immunotherapy is much less studied. Herein, we review the up-to-date findings on the influence of gut microbiota on three common immunotherapies including adoptive cell transfer, immune checkpoint blockade, and CpG-oligodeoxynucleotide therapy. We then explore three microbiota-targeted strategies that may improve treatment efficacy, involving dietary intervention, probiotics supplementation, and fecal microbiota transplantation.

## Introduction

The human gastrointestinal tract harbor thousands of microbial species. For example, intestines consist of a dense community with around 10^13^ microbes mainly from phyla *Bacteroidetes* and *Firmicutes*;^1^ while microbial abundance in the stomach is the least along the tract due to its extreme acidity with predominant expression of *Firmicutes* and *Proteobacteria*.^2^ These microorganisms form a microbiota (referring to an ecological community of microbes that is found within a specific environment), which interacts with a variety of host cells to contribute physiological functions including nutrient metabolism and gut barrier regulation.^3,4^ In particular, the gut microbiota substantially contributes to immune homeostasis as exemplified by using germ-free animals (referring to animals raised in the strict sterile conditions that have no microbes living in/on them), which displayed impaired development of regulatory T cells (T_Reg_) and poor growth of gut-associated lymphoid tissues.^3–5^ Whereas the host immunity can, in turn, manipulate the microbial profile: secretary immunoglobulin-A (IgA) from gut plasma cells has reactivity to a broad spectrum of microbes, and these IgA could enhance translocation of selected commensals into lymphoid tissues to facilitate antigen presentation and regulate microbial diversity.^6–8^

**Figure 1.**
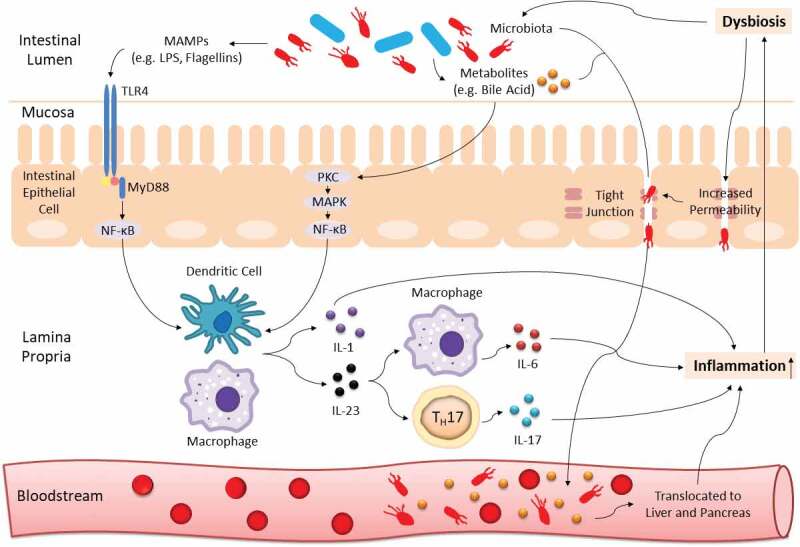
Gut dysbiosis interacts with host immunity to induce chronic inflammation. Blue and red rods represent beneficial commensals and pathobionts respectively. TLR4 from innate immunity recognizes MAMPs (e.g. LPS, flagellins) of dysbiotic microbiota and leads to initiation of NF-κB-dependent downstream production of pro-inflammatory cytokines (e.g. IL-1, IL-6, IL-17, IL-23). The NF-κB signaling cascade can also be activated by microbial derivatives especially a group of metabolites known as bile acid. In addition, dysbiosis can increase gut barrier permeability to induce translocation of pathobionts and metabolites from the mucosa to bloodstream, and eventually into the hepatopancreatic ductal system. All these processes can cause persistent inflammation, which can further exaggerate the imbalanced microbial community, thus forming a vicious cycle and promoting carcinogenesis

The immunity-microbiota crosstalk is continuously regulated in a healthy state. Yet, such equilibrium is readily affected by host genetic background and numerous environmental factors especially dietary intake.^9–11^ Once the extrinsic force overpowers the intrinsic stability, dysbiosis, termed as a compositional and functional alteration in the microbiota, can occur.^12^ With aid of the next-generation microbial sequencing, it is now well established that a shift in microbiota profile is greatly associated with cancer development and progression,^13,14^ and tremendous work has been conducted to decipher the underlying mechanism. Briefly, enriched microbe-associated molecular patterns (MAMPs; molecules that are found in/on microbes, e.g. flagellins, lipopolysaccharides (LPS), and can be recognized by recognition receptors of the innate immune system) trigger enhanced toll-like receptor (TLR)-mediated immune response which leads to inflammation. While persistent inflammation can exacerbate the imbalanced microbial community, thus forming a vicious loop and eventually resulting in gastrointestinal carcinogenesis^14–16^ (an illustration on this interaction is shown in Figure 1).

Given its pivotal role in gastrointestinal cancer, it is plausible to target the gut microbiota as a therapeutic strategy. Indeed, accumulated studies have illustrated that the commensal microbes can impact the efficacy of chemotherapy.^17^ In comparison, evidence on how microbiota correlates with immunotherapy only emerges recently. Here we summarize and discuss the up-to-date findings on the association of gut microbiota with most-studied immunotherapies including adoptive cell transfer (ACT), immune checkpoint blockade, and CpG-oligodeoxynucleotide therapy. Also, in acknowledgment of a relatively deeper understanding of the association between microbiota and gastrointestinal cancer than other cancers, we highlight three microbiota-targeted strategies that may improve treatment efficacy against gastrointestinal cancer, involving dietary intervention, probiotics, and fecal microbiota transplantation (FMT).

## Gut microbiota and immunotherapy

Resistance to traditional therapies including chemotherapy and radiotherapy is associated with high tumor recurrence and has been the key problem in curing cancer patients.^18^ In the last decade a bloom of clinical trials has displayed the potential of immunotherapy in treating cancers, of which several FDA-approved strategies could provide long-lasting anticancer effects to patients who were unresponsive to conventional treatment. Meanwhile, apart from its role in carcinogenesis, the gut microbiota also demonstrates its influence across a range of cancer treatments.^19,20^ In the following section, we discuss the role of gut microbiota in three major immunotherapy approaches involving ACT, immune checkpoint blockade, and CpG-oligodeoxynucleotide therapy (summarized in Table 1).

**Table 1.** Summary of the association of gut microbiota with host immunity to impact cancer immunotherapy

Immuno-therapy	Influence on Therapy	Phylum	Bacterium	Host	Correlation with Host Immunity	Ref.
ACT	Enhancement	*Bacteroidetes*	*Bacteroides*, *Parabacteroides*	TB Mice	Raise systemic Cd8α+ DCs; Induce T_H_1-mediated immune response	^21^
		-	Gram-negative LPS-producing bacteria	TB Mice	Induce microbial translocation into lymph nodes to activate Tlr4 signaling for PIC production	^22^
	Regression	*Bacteroidetes*	*S24-7*	TB Mice	-	^21^
Anti-CTLA-4 Therapy	Enhancement	*Bacteroidetes*	*Bacteroides fragilis*	TB Mice	Induce T_H_1-mediated immune response and DC maturation in tumor-draining lymph nodes	^23^
		*Firmicutes*	*Faecalibacterium, Lachnospiraceae, Ruminococcus*	Melanoma Patients	Relate to low systemic T_Reg_, CD4+ and CD8 + T cells at baseline; Raise systemic CD4 + T cells upon treatment initiation	^24^
		*Proteobacteria*	*Burkholderia cepacia*	TB Mice	Induce T_H_1-mediated immune response and DC maturation in tumor-draining lymph nodes	^23^
Anti- PD-1/PD-L1 Therapy	Enhancement	*Actinobacteria*	*Bifidobacterium abreve*	TB Mice	Induce DC-mediated immune response, T cell activation and PIC production	^25^
			*Bifidobacterium adolescentis*	TB Mice	Induce DC-mediated immune response, T cell activation and PIC production	^25^
				Melanoma R	Raise tumor-specific CD8 + T cells	^26^
			*Bifidobacterium dentium*	HCC R	Relate to anti-inflammation	^27^
			*Bifidobacterium longum*	TB Mice	Induce DC-mediated immune response, T cell activation and PIC production	^25^
				Melanoma R	Raise tumor-specific CD8 + T cells	^26^
				NSCLC & RCC R	Raise systemic memory CD8 + T cells & NK cells	^28^
			*Enterococcus faecium*	Melanoma R	Raise tumor-specific CD8 + T cells	^26^
		*Bacteroidetes*	*Alistipes putredinis*, *Prevotella corpri*	NSCLC & RCC R	Raise systemic memory CD8 + T cells & NK cells	^28^
			*Parabacteroides merdae*	Melanoma R	Raise tumor-specific CD8 + T cells	^26^
		*Firmicutes*	*Faecalibacterium*	Melanoma R	Raise systemic and intratumoural effector CD4+ and CD8 + T cells	^29^
			*Lactobacillus*	Melanoma R	Raise tumor-specific CD8 + T cells	^26^
				HCC R	Inhibit inflammatory response by reducing oxidative stress injury	^27^
			*Ruminococcus obeum*^a^	HCC R	Inhibit inflammatory response by reducing oxidative stress injury	^27^
			*Veillonella parvula*	Melanoma R	Raise tumor-specific CD8 + T cells	^26^
		*Proteobacteria*	*Klebsiella pneumoniae*^b^	Melanoma R	Raise tumor-specific CD8 + T cells	^26^
		*Verrucomicrobia*	*Akkermansia muciniphila*	NSCLC & RCC R	Induce IL-12-dependent intratumoural infiltration of Ccr-9+ Cxcr-3+ Cd4 + T cells	^30^
				HCC R	Relate to anti-inflammation	^27^
	Regression	*Bacteroidetes*	-	Melanoma R	Raise systemic T_Reg_ & MDSCs	^29^
			*Bacteroides*	Melanoma Patients	Relate to high systemic CD4 + T cells at baseline; No increase in T cell induction upon treatment initiation	^24^
		*Firmicutes*	*Ruminococcus (obeum*^a^)	Melanoma, RCC & NSCLC NR	-	^26,28^
		*Proteobacteria*	*Escherichia coli, Klebsiella pneumoniae*^b^	HCC NR	-	^27^
ICI-Induced irAEs	Regression	*Actinobacteria*	*Bifidobacterium*	DSS-TB Mice	Induce T cell-related metabolism	^31^
		*Bacteroidetes*	Bacteroidaceae, Barnesiellaceae, Rikenellaceae	Colitis-Free Melanoma Patients	Induce polyamine transport and B vitamin biosynthesis	^32^
			*Bacteroides uniformis, Parabacteroides distasonis, Prevotella*	Melanoma Patients	Relate to high systemic CD4 + T cells at baseline; No increase in T cell induction upon treatment initiation	^24^
	Enhancement	*Firmicutes*	*Clostridiales bacterium, Faecalibacterium prausnitzii*	Melanoma Patients	Relate to low systemic T_Reg_, CD4+ and CD8 + T cells at baseline; Raise systemic CD4 + T cells upon treatment initiation	^24^
CpG-ODN Therapy	Enhancement	*Bacteroidetes*	*Alistipes shahii*	TB Mice	Induce TNF production by tumor-infiltrating lymphocytes and tumor-associated myeloid cells	^33^
		*Firmicutes*	*Ruminococcus*			
		-	Gram-negative LPS-producing bacteria			
	Regression	*Firmicutes*	*Lactobacillus*	TB Mice	-	^33^

^a^Contradictory findings as being correlated with both enhancement and regression of the efficacy of anti-PD-1/PD-L1 therapy.

^b^Contradictory findings as being correlated with both enhancement and regression of the efficacy of anti-PD-1/PD-L1 therapy.

*Abbreviations*: DC, Dendritic cells; NR, Nonresponders; PIC, Pro-inflammatory cytokines; R, Responders; TB, Tumor-bearing.

### Adoptive immune cell transfer

ACT is a treatment approach to utilize autologous immune cells such as tumor-infiltrating lymphocytes (TILs) and cytotoxic T lymphocytes (CTLs) against cancer. It includes three steps: (1) isolating T cells from tumor tissues or peripheral blood vessels; (2) co-culturing with interleukin (IL)-2 to allow *ex vivo* expansion; and (3) reinfusion of extracted T cells back into the patients.^34,35^ Whereas most recent clinical trials involves genetically modified to enhance expression of antigen-specific T cell receptor (TCR) or chimeric antigen receptor (CAR) on extracted T cells, thereby triggering stronger anticancer immunological response after reinfusion to overcome immuno-resistant mechanisms of tumor cells (e.g. defective antigen processing).^34–36^ ACT has shown a greater specificity than chemotherapy since autologous immune cells are used and could be genetically modified to recognize and target specific tumor antigens; thus, ACT is considered as a highly personalized cancer therapy.^37,38^ To date ACT especially CAR-T cell therapy has displayed remarkable success in eradicating hematologic malignancies^36,39^ and metastatic melanoma.^40,41^ While the efficacy of ACT in gastrointestinal tumors including colorectal cancer (CRC),^42^ hepatocellular carcinoma (HCC),^34^ and esophageal cancer^43,44^ is limited, which may be attributed to poor trafficking to tumors, dysregulated T_Reg_/effector T cell ratio, and immunosuppressive tumor microenvironment.^45,46^ Extensive work is ongoing to optimize ACT by modulating tumor-immune microenvironment (TIM),^46^ modifying genetic engineering strategies,^47^ or coupling with radiotherapy.^48^ Arising trials are developing alternatives such as CAR-expressing nature killer (NK) cell therapy,^49^ and combining dendritic cells and cytotoxic-induced killer cells to treat HCC and pancreatic cancer.^50–52^

#### Gut Microbiota and Adoptive Immune Cell Transfer

The first evidence showing the correlation of gut microbiota with ACT efficacy was reported in 2007 when ACT plus total body irradiation (TBI; a form of lymphodepletion) were applied to a mouse model with a deficiency in a cluster of differentiation (Cd)-14 and Tlr-4.^22^ Depleting microbiota by antibiotics or suppressing LPS signaling components impaired the function of adoptively transferred Cd8 + T cells and decreased the number of activated dendritic cells, resulting in reduced anticancer efficacy. Whereas LPS administration to TBI-treated microbiota-depleted mice could promote proliferation and function of reinfused T cells, and even be able to long-term cure mice with large tumors. Mechanically, TBI could induce microbial translocation particularly gram-negative LPS-producing bacteria into the mesenteric lymph nodes. These translocated microbes then initiate the Tlr4 pathway by expressing various Tlr4 agonists (e.g. LPS, peptidoglycan), leading to enhanced activation of dendritic cells with increased secretion of pro-inflammatory cytokines including Il-1β, Il-6, and tumor necrosis factor (Tnf)-α across the gut. Notably, LPS administration could improve anticancer response mediated by adoptively transferred Cd8 + T cells in TBI-untreated mice.

In 2018 Uribe-Herranz *et al*. applied ACT to tumor-bearing C57BL/6 mice fromtwo vendors (JAX and HAR) and found that tumor growth is almost completely abolished in HAR mice but not JAX mice.^21^ The 16S rRNA sequencing revealed the difference in composition of fecal microbiota between JAX and HAR mice – a diverse range of *Bacteroidetes* taxa was present in HAR mice, while JAX mice was dominated by *Bacteroidales S24-7*, hence suggesting that ACT efficacy may correlate to several genera of *Bacteroidetes* including *Bacteroides* and *Parabacteroides*. An antibiotic vancomycin was then used to eliminate the gram-negative phylum *Bacteroidetes* in JAX and HAR mice. In terms of efficacy, no change was observed in HAR mice but tumor regression in JAX mice was greatly enhanced to match the impact of ACT on HAR mice without vancomycin treatment. Such amelioration was attributed to enhanced T helper cell (T_H_)-1-mediated immune response, and accumulation of peripheral Cd8α+ dendritic cells, resulting in increased expansion and activity of adoptively transferred T cells. Whilst no phenotypic change was observed under antibiotic treatments of neomycin and metronidazole, thus implicating the role of specific bacteria in mediating host response to ACT. Collectively, these findings suggest a potential way to enhance response to ACT by altering the gut microbiota, yet it remains elusive that which specific microbes are responsible for such improvement. An in-depth mechanistic study is, therefore, necessary to identify reliable microbial targets for modulating the ACT efficacy before proceeding to clinical trials.

### Immune checkpoint blockade

The aim of immune checkpoint blockade is to restore and strengthen the anticancer response by suppressing the intrinsic immuno-inhibitory pathways, which are commonly utilized by tumor cells to develop immune resistance.^35^ Enormous efforts have been invested to exploit the efficacy of treating cancer patients with fully-humanized monoclonal antibodies against two of the most-studied immune checkpoint regulators – cytotoxic T lymphocyte-associated antigen-4 (CTLA-4) and programmed cell death protein-1 (PD-1) or its ligand PD-1-ligand 1 (PD-L1). Both CTLA-4 and PD-1 are TCRs belonging to the immunoglobulin superfamily,^53,54^ but they share different features and mechanisms in regulating host immunity (Supplementary Table 1). To date, some immune checkpoint inhibitors (ICIs) have received FDA approval including blockers of PD-1 (nivolumab, pembrolizumab, and cemiplimab), PD-L1 (atezolizumab, avelumab, and durvalumab), and CTLA-4 (ipilimumab) for treating cancers, particularly metastatic melanoma, and nonsmall-cell lung cancer (NSCLC; Supplementary Table 2). These ICIs can also be used against several gastrointestinal cancers involving HCC,^55,60^ gastric cancer,^57,58^ esophageal carcinoma,^59^ and DNA mismatch repair-deficient or microsatellite instability-high (dMMR/MSI-H) CRC^56,61^ (Table 2). However, ICI therapy is frequently linked with immune-related adverse events (irAEs) such as colitis and pneumonitis.^63,64^ Arising evidence has now revealed the correlation between irAEs incidence and efficacy of ICI therapy.^65,66^ Together with high variation in therapeutic responsiveness (45–60% for patients with melanoma or MSI-H tumors; and 15–30% for patients with solid tumors^66^), it is thus critical to develop strategies to reduce the occurrence of irAEs and enhance treatment efficacy.

**Table 2.** FDA-approved immune checkpoint blockade against gastrointestinal cancer

Target	Drug Name	Brand Name	Indication for GIC^a^	Reference
PD-1	Nivolumab	Opdivo	HCC dMMR/MSI-H CRC	NCT01658878^55^ NCT02060188^56^
	Pembrolizumab	Keytruda	GC ESCC HCC dMMR/MSI-H CRC	NCT02335411;^57^ NCT02370498^58^ NCT03189719^59^ NCT02702414^60^ NCT02054806^61^
	Cemiplimab	Libtayo	-	-
PD-L1	Atezolizumab	Tecentriq	-	-
	Avelumab	Bavencio	-	-
	Durvalumab	Imfinzi	-	-
CTLA-4	Ipilimumab	Yervoy	-	-
Combined	Nivolumab plus Ipilimumab	Opdivo & Yervoy	dMMR/MSI-H CRC	NCT02060188^56,62^

^a^Unless further specification, all included indications are applied as monotherapy. *Abbreviations*: ESCC, Esophageal squamous cell carcinoma; GC, Gastric cancer.

Given that CTLA-4 and PD-1 regulate immune response through distinct mechanisms, a combination of anti-CTLA-4 and anti-PD-1/PD-L1 drugs were therefore proposed to improve patient outcomes. An early animal study revealed that anti-CTLA-4 antibodies could act synergistically with PD-1 blockade to increase effector T cell infiltration and allow continuous expansion of tumor-specific T cells, thereby shifting TIM from suppressive to inflammatory.^67^ Currently, there is one FDA-approved combination – nivolumab (3 mg/kg) plus low-dose ipilimumab (1 mg/kg) for treating metastatic melanoma, renal cell carcinoma (RCC; Supplementary Table 2), and dMMR/MSI-H CRC (Table 2). Most joint-ICI therapies yield more positive results in clinical trials with less side effects when comparing with monotherapy except for pembrolizumab-ipilimumab combined treatment, which showed high toxicity in melanoma patients.^68,69^ In dMMR/MSI-H CRC, the objective response rate and 12-month overall survival in patients treated with both nivolumab and ipilimumab increased by 24% and 12%, respectively, when comparing with patients receiving nivolumab only (NCT02060188,^56,62^). Similar results were reported when applying joint-ICI therapy with an uncommon dosage (1 mg/kg of nivolumab plus 3 mg/kg of ipilimumab) on patients with chemotherapy-refractory gastroesophageal cancer, of which the objective response rate, and 12-month progression-free survival increased by 12% and 9%, respectively, together with 30% decrease in the incidence of grade 3–4 treatment-related adverse events (NCT01928394,^70^). Other novel approaches to improve the safety and efficacy of ICI therapy include combination with neoantigen vaccines, chemotherapy, or other anticancer drugs, as well as modulating the gut microbiota profile.^68,69^ In addition, Conforti et al. conducted a dedicated meta-analysis on a total of 11,351 cancer patients from 20 clinical trials to decipher whether gender difference can influence immune checkpoint blockade.^71^ Their findings revealed that although both ICI therapy significantly improves overall survival in patients of both sexes, the magnitude of this benefit is largely sex-dependent with men showing much greater efficacy than women. Extensive work is therefore needed to improve treatment outcomes for women or perhaps designing differential immunotherapeutic approaches between men and women.

#### Gut microbiota and immune checkpoint blockade – preclinical studies

##### CTLA-4 blockade

In 2015 Vétizou et al. found that a significant decrease in activated effector Cd4 + T cells and TILs are occurred in tumor-bearing mice treated with broad-spectrum antibiotics or housed in germ-free conditions, resulting in ineffective CTLA-4 blockade.^23^ Reduction of *Bacteroidales* and *Burkholderiales* in the faces of these microbiota-depleted mice was identified. Notably, re-colonization of species from these two taxa including *Bacteroides thetaiotaomicron, Bacteroides fragilis*, and/or *Burkholderia cepacia* into microbiota-depleted mice rescued CTLA-4 blockade resistance by promoting T_H_1-mediated immune response and dendritic cell maturation in tumor-draining lymph nodes, meanwhile alleviating anti-CTLA-4-induced colitis. Adoptive transfer of *B. fragilis*-specific T_H_1 cells could also restore sensitivity to CTLA-4 blockade. In another study, vancomycin supplementation to mice before administration of anti-CTLA-4 antibodies and dextran sulfate sodium (a colitogen to model blockade-induced colitis) provoked a more severe and largely fatal form of the disease, implicating the role of gram-positive bacteria in mitigating CTLA-4 blockade-induced colitis.^31^ Of note, oral gavage of a mixture of four gram-positive *Bifidobacterium* species could ameliorate the immunopathology associated with CTLA-4 blockade by upregulating T cell-mediated metabolic processing, thereby rescuing mice from vancomycin-induced dysbiosis.

##### PD-1/PD-L1 blockade

In 2015 Sivan *et al*. compared the growth of subcutaneous melanoma between C57BL/6 mice obtained from two vendors (JAX and TAC) which are known to differ in their commensal microbes.^25^ They revealed that tumors in TAC mice grow faster and are less sensitive to anti-PD-L1 antibodies compared with JAX mice, and these differences were associated with lower intratumoral infiltration of Cd8 + T cells and weaker tumor-specific T cell response. When cohousing with JAX mice or administering feces from JAX mice, TAC mice acquired the phenotypes as observed in JAX mice with improved responsiveness to PD-L1 blockade to an extent similar to anti-PD-1-treated JAX mice. The 16S rRNA sequencing on fecal samples of JAX-fed TAC mice identified that *Bifidobacterium* species including *Bifidobacterium adolescentis, Bifidobacterium breve*, and *Bifidobacterium longum* showed the largest increase in abundance and strongest association with anticancer T cell response. Of note, oral gavage of a commercially available cocktail of *Bifidobacterium* spp. involving *B. breve* and *B. longum* to TAC mice was sufficient to improve dendritic cell-mediated immune responses (e.g. increased level of interferon-γ, accumulation of peripheral tumor-specific T cells, and intratumoral Cd8 + T cells), leading to ameliorated tumor control to the same extent as anti-PD-L1 antibodies, whereas combining both treatments almost abolished all tumor growth. Collectively, these data indicates that commensal *Bifidobacterium* could influence host anticancer immunity, thereby enhancing the efficacy of PD-1/PD-L1 blockade.

#### Gut microbiota and immune checkpoint blockade – microbial profiling studies

Numerous studies have utilized next-generation sequencing to investigate the correlation between gut microbiota and therapeutic response in patients treated with PD-1/PD-L1 blockade by comparing the diversity and composition of fecal microbiota in responders with nonresponders. Bacterial species enriched in responders and their corresponding mechanisms are listed in Table 1. Some of these species include *B. longum, Collinsella aerofaciens*, and *Enterococcus faecium* in metastatic melanoma ;^26,29^ and *B. longum, Akkermansia muciniphila*, and *Prevotella corpri* in NSCLC and RCC.^28,30^ To establish a cause–effect relationship between commensals and blockade efficacy, feces from responding patients were transplanted into tumor-bearing mice with microbiota depleted by either antibiotics or housing in germ-free condition.^26,29,30^ The efficacy of PD-1/PD-L1 blockade was ameliorated in these recipient mice in relation to enhanced T cell response and anticancer immunity, whilst transplantation of feces from nonresponders failed to do so. Notably, administration of *A. muciniphila* to microbiota-depleted mice with fecal transplantation from nonresponders stimulated Il-12-dependent infiltration of Ccr-9+ Cxcr-3+ Cd4 + T cells into tumor beds, resulting in the restoration of the anticancer effect of PD-1 blockade.^30^

In comparison, there were much fewer investigations on how the gut commensals influence response to ICI therapy in patients with gastrointestinal cancers. Zheng *et al*. prospectively analyzed the fecal samples of HCC patients receiving an anti-PD-1 drug camrelizumab.^27^ Before treatment, the fecal microbiota in both responders and nonresponders was dominated by *Bacteroidetes* following by *Firmicutes* and *Proteobacteria*, which are in accordance with findings in healthy adults.^1^ When treatment proceeded, *Proteobacteria* species including *E*scherichia *coli* and *Klebsiella pneumoniae* markedly increased in nonresponders, while microbial composition in responders remained stable. Subsequent analysis identified 20 significantly enriched species in responders and their associated functional pathways, including *Bifidobacterium dentium*, correlated with anti-inflammatory cellulose metabolism; four *Lactobacillus* species, correlated with reducing oxidative stress injury; and two *Ruminococcaceae* species and *A. muciniphila*, which were reported capable of improving anti-PD-1/PD-L1 efficacy,^29,30^ were correlated with multiple critical metabolisms. Overall, these findings illustrate that the gut commensals are closely related to patient responsiveness to ICI therapy. Targeting or modulating the microbiota to manipulate its composition could therefore be a potential clinical strategy to enhance therapeutic response. It is noteworthy that to date there is insufficient profiling work on revealing the correlation between microbiota and efficacy of joint-ICI immunotherapy.

#### Gut microbiota and immune checkpoint blockade – clinical studies

##### Antibiotic treatment

It is common for clinicians to prescribe broad-spectrum antibiotics to patients with prolonged immunosuppression to prevent opportunistic infections, and patients with immune checkpoint blockade-induced diarrhea accompanied by fever or leukocytosis.^72,73^ However, as antibiotics are well known to cause compositional alteration in the gut microbiota, several clinical trials were conducted to depict the impact of antibiotics on patients receiving ICI therapy. Derosa et al. performed the largest independent retrospective study by far to assess the effect of antibiotic treatment prior to the first dose of the anti-PD-1/PD-L1 drug in 360 patients with RCC (*n* = 121) or NSCLC (*n* = 139).^74^ Shorter progression-free survival (PFS) and overall survival was observed in RCC patients exposed to antibiotic 30 or 60 d as well as NSCLC patients exposed to antibiotics 30 d before treatment initiation. Whereas in the retrospective study of Sen et al. involving 172 patients with RCC, NSCLC, melanoma, sarcoma, and gastrointestinal stromal tumors, antibiotics were applied to patients during (*n* = 54), 30 (*n* = 19) or 60 d (*n* = 14) prior to PD-1 and/or CTLA-4 blockade.^75^ Contrastingly, neither PFS nor overall survival showed any difference between antibiotic-treated and untreated patients at all time points, except that a decrease in overall survival was observed in patients with the use of antibiotics 30 d before treatment. Similar results were obtained in another retrospective study involving 161 patients with gastroesophageal cancer.^76^ To elucidate the heterogeneity among studies, Huang et al. conducted a dedicated meta-analysis to pool all data from 19 relevant publications comprising a total of 2,740 cancer patients.^77^ Statistically significant reduction in PFS and overall survival were observed when comparing blockade-treated patients with the use of antibiotics to those without regardless of the cancer type, thus indicating the negative association between antibiotics and efficacy of ICI treatment.

Further evaluation on how the initiation time of antibiotic treatment impact patient outcomes was done by Pinato et al.^78^ Broad-spectrum antibiotics were given to 29 and 68 patients (malignant melanoma or NSCLC) 30 d before or during anti-PD-1/PD-L1 therapy, respectively. Only pretreatment use of antibiotics but not concurrent use was associated with worse overall survival and a higher risk of primary disease refractory, suggesting that antibiotic application is still safe for patients who are undergoing ICI therapy. Nevertheless, these clinical work has revealed the proof-of-concept relationship between microbiota and antibiotics as demonstrated preclinically. Although the result has been controversial, it is obvious that antibiotics have no significant benefits or even worsens treatment responsiveness. Extensive work is thus required to ensure the safety and necessity before prescribing antibiotics to patients who would receive immune checkpoint blockade.

##### Blockade-induced adverse events

ICI therapy could induce various irAEs such as colitis and hepatitis in the gastrointestinal tract.^63,64^ A prospective study in 2016 identified an increased fecal abundance of three *Bacteroidetes* families (*Bacteroidaceae, Barnesiellaceae*, and *Rikenellaceae*) in 24 ipilimumab-treated melanoma patients who did not develop blockade-induced colitis.^32^ Later in 2019, a similar study also reported the enriched fecal abundance of *Bacteroides* and *Parabacteroides* from phylum *Bacteroidetes* in 18 pembrolizumab-treated lung cancer patients without the development of blockade-induced diarrhea.^79^ In a retrospective study involving 826 patients with ICI therapy, the use of antibiotics no matter before or after treatment was associated with reduced occurrence and recurrence of irAEs.^80^ Yet additional hospitalization and immunosuppressive treatment (e.g. corticosteroid supplementation) were more often needed for those receiving antibiotics after ICI therapy. Of note, antibiotics administrated at the onset of irAEs were correlated with enhanced severity and recurrence of irAEs. These clinical data thus illustrates that alteration in the microbial profile is associated with irAE incidence in patients treated with immune checkpoint blockade. Although antibiotics seem to be effective in preventing the onset of irAEs, depleted microbiota could meanwhile increase the occurrence of more severe irAEs.

In 2017 Chaput et al. accessed the composition of fecal microbiota at baseline and during ipilimumab treatment in a prospective cohort with 26 metastatic melanoma patients.^24^ Patients whose baseline microbiota enriched with *Ruminococcus* and *Faecalibacterium* had longer PFS and overall survival, whilst the high proportion of *Bacteroides* was present in patients with poorer clinical benefits. Contrastingly patients with baseline enrichment of *Firmicutes* species (e.g. *Clostridiales bacterium* and *Faecalibacterium prausnitzii*) were more likely to develop blockade-induced colitis, while the fecal microbiota in colitis-free patients was dominated by *Bacteroidetes* (e.g. *Prevotella* and *Bacteroides uniformis*), which is in accordance with previous findings.^32,79^ Low baseline levels of systemic pro-inflammatory cytokines, CD4+, and CD8 + T cells, and a substantial increase in CD4 + T cells upon treatment initiation were observed in patients with *Faecalibacterium*-dominant fecal microbiota who showed long-term clinical benefits but a higher incidence of colitis. For patients with *Bacteroides*-dominant fecal microbiota who had a lower occurrence of colitis but instead with poorer therapeutic response, a much higher proportion of CD4 + T cells at baseline and no enhanced T cell induction after treatment start was observed, thus indicating that the microbial profile at baseline could predict patient outcome and toxicity to ICI therapy. Taken together, these findings demonstrate the correlation between microbiota and immune checkpoint blockade as well as its related adverse events. Given that modulating the commensals directly to ameliorate treatment efficacy is yet to show the convincing results in practice, targeting the microbiota composition and utilizing it as a prediction tool for the patient outcome may instead yield a promising direction.

### Gut microbiota and CpG-oligodeoxynucleotide therapy

Unmethylated CpG dinucleotide motif originally exists in a bacterial genome. It is a MAMP that could trigger the host immunity to initiate TLR9/IL-1 R-mediated signaling cascade, leading to upregulation of pro-inflammatory cytokines, and activation of IRF and NF-κB downstream pathway.^81,82^ Synthetic oligodeoxynucleotides (ODNs) comprising of CpG motifs similar to those naturally found in bacteria but with less toxicity have been developed, and these CpG-ODNs could be recognized by myeloid and dendritic cells to stimulate immune activation.^83,84^ Several CpG-ODNs especially CpG-7909 has been applied in clinical trials.^85^ However, unimpressive results were often revealed as monotherapy seemed to be insufficient to induce robust anticancer effect, which could be explained by distinct expressing patterns of TLR9 among patients, and overshadowing the immuno-stimulatory effect of CpG-ODNs by the immunosuppressive tumor microenvironment.^86^ Meanwhile, large efforts have been invested to apply CpG-ODNs as an adjuvant of other treatments including chemotherapy and immunotherapy.^86,87^

Preclinically, intratumoural or peritumoral injection of CpG-ODNs plus ICIs increased the circulating levels and tumoral infiltration of effector Cd8 + T cells, thereby prolonging the survival of tumor-bearing blockade-resistant mice.^88,89^ In the study of Wang et al., two mouse models that mimic anti-PD-1-resistance as inpatients were developed.^90^ A synergistic effect was observed when CpG-ODN SD-101 plus anti-PD-1 antibodies were injected intratumorally SD-101 altered TIM by promoting T cell infiltration and generation of multifunctional Cd8 + T cells, and subsequent PD-1 blockade led to further expansion of CpG-induced Cd8 + T cells differentiating into short-lived effector cells and long-lived memory precursors. These results thus provide a rationale for proceeding into trials with the use of this innovative CpG-ODN-immune checkpoint blockade-combined strategy.^86^

In germ-free mice or antibiotic-treated mice, injection of CpG-ODNs and anti-IL-10 R antibodies failed to reduce subcutaneous tumor growth with shortened survival when compared to those without microbiota depletion.^33^ Reduced secretion of pro-inflammatory cytokines (Il-1α, Il-1β) and lowered Cd45+ TILs-produced Tnf was occurred in these microbiota-depleted mice after injection. Ineffective treatment was also observed in *Tlr4*^−/-^ mice, whilst the administration of Tlr4 agonist LPS could restore the responsiveness of myeloid cells toward treatment in wild-type mice with impaired microbiota. This indicates that gut microbiota could prime tumor-associated myeloid cells through Tlr4 activation to provoke Tlr9-dependent immune response upon CpG-ODN injection. Several fecal bacteria were correlated with CpG-ODN efficacy, of which gram-negative (e.g. *Ruminococcus* and *Alistipes shahii*) and gram-positive (e.g. *Lactobacillus fermentum, Lactobacillus intestinalis*, and *Lactobacillus murinum*) genera were positively and negatively correlated to CpG-ODN-induced Tnf production, respectively. Notably, when administrating *A. shahii* to microbiota-depleted CpG-ODN-treated mice, the ability of tumor-associated myeloid cells to produce Tnf was restored. In contrast, oral gavage of *L. fermentum* (well-established anti-inflammatory species^91,92^) attenuated anticancer response to CpG-ODNs in mice pretreated with antibiotics. Altogether it is now clear that the efficacy of CpG ODNs or other immunotherapies is closely related to the commensal microbiota. As different microbes could lead to opposing therapeutic responses, altering the microbial community by clinical interventions such as probiotics and FMT could be a feasible approach to further ameliorate the anticancer effect of cancer treatments.

## Targeting gut microbiota as adjuvants of immunotherapy

**Figure 2.**
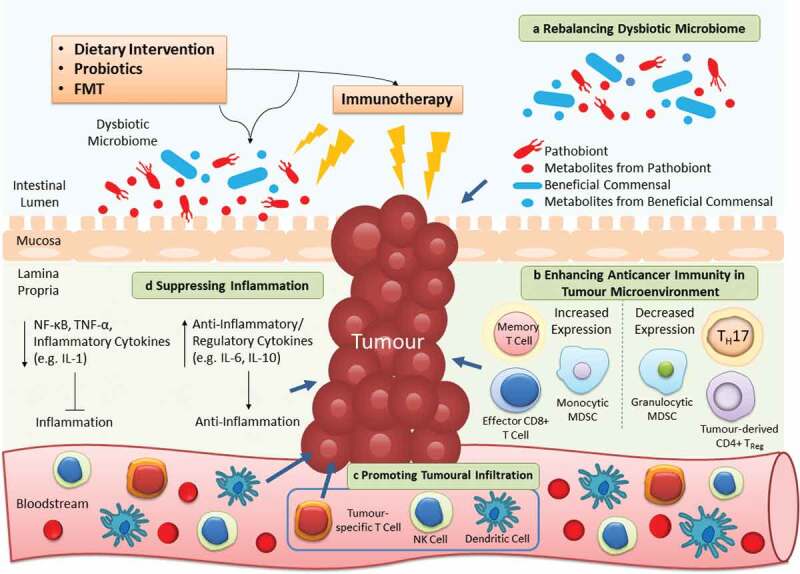
Targeting gut microbiota as adjuvants of cancer immunotherapy. Dietary intervention, probiotics and FMT are microbiota-targeted strategies that can ameliorate the efficacy of immunotherapy in 4 distinct mechanisms. a | As the microbiota composition is easily affected, utilizing these extrinsic strategies can restore the imbalanced microbial community to alleviate dysbiosis-associated pathology. b | The anticancer immunity in tumor microenvironment is inhibited to flavor tumor cell growth. By reconstructing the T cell repertoire, the suppressed host immunity can be provoked once again to fight against cancer. c | A diversity of immune cells (e.g. NK and dendritic cells) infiltrate from the circulation into the tumor to further contribute to killing of cancer cells. d | Apart from direct effects on the tumor, the anticancer immunity is stimulated by these strategies to increase or decrease production of anti-inflammatory or pro-inflammatory cytokines respectively, thereby alleviating persistent inflammation in cancer patients

Given its therapeutic potential, growing interest in targeting the gut microbiota to alleviate dysbiosis or associated inflammation, and utilizing it as adjuvants of immunotherapy have been arisen (relevant ongoing clinical trials are listed in Table 3). Here we discuss three strategies involving dietary intervention, probiotics and FMT that aim to alter the microbial community to contribute greater clinical benefits to patients treated with immunotherapy, which is summarized in Figure 2.

**Table 3.** Ongoing clinical studies on the role of microbiota in gastrointestinal cancer.^a.^

Study Type	Trial Information	Study Model	Time Perspective	Patient Population	Intervention	Study Aim
Observational	NCT01313442; US; R	Cohort	Prospective	GIC (*n* = 500)	-	Cohort establishment
	NCT02726243; France; R	Case-Control	Prospective	Healthy, IBD, CRC (*n* = 240)		
	NCT03998644; China; R	Case-Control	Cross-Sectional	Healthy, CRA, CRC (*n* = 2000)		
	NCT04015466; EU & CELAC; R	Cohort	Prospective	GC (*n* = 800)		
	NCT04189393; Netherlands; NR	Cohort	Prospective	GIC (*n* = 60)		Map the oral and gut microbiome in patients
	NCT03623152; Hong Kong; R	Case-Only	Cross-Sectional	Healthy, CRA, CRC (*n* = 160)		Compare microbiota in left and right colon
	NCT03841799; France; R	Cohort	Prospective	CRC (*n* = 80)		Study gut microbiota and immune infiltration
	NCT03385213; China; R	Case-Control	Retrospective	Patients with relapse CRC (*n* = 200)		Study gut microbiota in patients with relapse CRC
	NCT03667495; China; R	Other	Prospective	Patients with relapse CRC (*n* = 100)		Study gut and oral microbiota in patients with relapse CRC
	NCT04005118; France; R	Cohort	Prospective	CRC (*n* = 50)		Study association of gut microbiota with post-operative complications
	NCT04071964; Canada; R	Other	Prospective	CRC (*n* = 300)		Study association of gut microbiota with healing after surgery
	NCT03191110; Netherlands; R	Cohort	Prospective	CRC (*n* = 2000)		Study association of lifestyle factors with CRC survival and recurrence
Interventional; Early Phase 1	NCT04130763; China; R	Single Group Assignment	-	Anti-PD-1 resistant or refractory GIC (*n* = 5)	2-week oral FMT from donors with similar microbiota as in anti-PD-1 responders	Test whether FMT can improve efficacy in anti-PD-1 resistant or refractory patients
Interventional; Phase 2	NCT03359681; Denmark; R	Parallel Assignment		CRC (*n* = 48)	Metformin HCL 20 d before and 10 d after surgery or placebo	Test the drug effects on tumor cell growth, immunological and metabolic change in CRC patients
	NCT03661047; US; R	Parallel Assignment		CRC (*n* = 36)	2-year daily intake of marine omega-3 fatty acid (4-gram) with treatment of AMR101 or placebo	Test the drug effects on TIM and microbiome; Examine the drug effects on tumor pathologic and molecular features prior to any other therapies
	NCT03831698; US; R	Singe Group Assignment		CRC, Lynch Syndrome (*n* = 34)	12-month daily intake of omega-3 fatty acid ethyl esters (2-gram)	Test the drug effects on molecular and microbiota changes in patients
	NCT03781778; US; R	Parallel Assignment		Stage I to III CRC survivors (*n* = 24)	8-week intake of resistant starch or regular corn starch as control	Test the dietary effects on inflammation, insulin resistance and microbiota in survivors
Interventional; Not Applicable	NCT03028831; US; R	Single Group Assignment		Healthy, CRA (*n* = 60)	4-week intake of resistant starch or digestible starch as control	Test the dietary effects on lowering CRC risk, adenoma recurrence and inflammation

^a^Clinical studies included were searched from clinicaltrials.gov using the following 2 key words: “gastrointestinal cancer” and “microbiota”. Completed trials were excluded, as well as investigations on the relationships between microbiota and chemotherapy/radiotherapy due to their irrelevancy to this article. The search was conducted in April 2020.

*Abbreviations*: CRA, Colorectal adenoma; GIC, Gastrointestinal cancer; HCL, Hydrochloride; NR, Not yet recruiting; R, Recruiting.

### Dietary intervention

Long-term-imbalanced diet has been correlated with cancer, for instance, food intake with insufficient fiber and excessive proteins from red meats is adequate to promote CRC development.^93–95^ Meanwhile, dietary nutraceuticals can prevent carcinogenesis as reported in thousands of work.^93,95,96^ Recently utilizing nutraceuticals especially polyphenols (a group of natural plant-derived chemicals) to treat cancer has been emerged. For example, resveratrol in grapes could enhance anticancer immunity in tumor-bearing mice by making TIM unfavorable for tumor growth (e.g. promoting accumulation of effector Cd8 + T cells and monocytic myeloid-derived suppressor cells (MDSCs), and inhibiting the population of tumor-derived Cd4+ Cd25+ T_Reg_ and Cd8 + T cell-suppressing granulocytic MDSCs^97–99^). When providing resveratrol to immunotherapy-treated tumor-bearing mice, complete tumor abolishment and metastasis retardation was observed without causing therapy-induced injury in normal epithelial cells.^100,101^ To date, there are no trials testing the efficacy of resveratrol-adjuvant immunotherapy, while short-term resveratrol administration (≤14 d) to CRC patients could reduce tumor cell proliferation^102^ and induce apoptosis (NCT00920803,^103^). Other polyphenols including flavonoids,^104^ genistein,^105^ and pomegranate^106^ have shown convincing anticancer activities in preclinical but not in clinical studies, which is suggested due to the unrealistic amount of nutraceuticals required for exhibiting some effects in patients.^107^ For instance, although curcumin is one of the most promising polyphenols as a potential adjuvant of CRC treatment, clinical use is often restricted due to its low water solubility and bioavailability.^107,108^ Several nano-formulations such as liposomes and micelles are thus developing to improve curcumin delivery into patients.^109^

A high-fat low-protein/carbohydrate dietary combination known as the ketogenic diet is well established for its neuroprotective effect against several neurological disorders including epilepsy and Alzheimer’s disease.^110^ Lowered glucose intake in a ketogenic diet can restrict tumor cell metabolism without affecting normal cells, as tumor cells rely on glucose as an energy source and are unable to metabolize ketone bodies.^110^ Whereas normal cells can metabolize both; thus, avoiding tumors to obtain sufficient energy. A ketogenic diet can also influence the host immunity: it suppresses lactate production by glycolytic tumors, leading to the enhanced anticancer immune response by inhibiting lactate-mediated tumoral immunosuppression and MDSC expression in tumor-bearing mice.^111^ To date, ketogenic diet intervention has shown varied anticancer efficacy in clinical trials,^112,113^ whereas combination with chemotherapy and radiotherapy has yielded more convincing results.^110^ While the efficacy of combining a ketogenic diet with immunotherapy is yet to be determined.

### Probiotics

Introducing exogenous probiotics with functional colonization can reward health benefits as exemplified by using probiotics to treat IBD.^114,115^ Regarding to cancer, apart from the well-known *Lactobacillus rhamnosus* GG which can inhibit expressions of inflammatory proteins NF-κB-p65, COX-2, and TNF-α to reduce colon tumor incidence,^116,117^ some *Lactobacillus* species also display immunomodulatory features to suppress carcinogenesis. *Lactobacillus casei* BL23 could improve immune response by reducing T_Reg_ level in mice with colitis-associated cancer;^118^ and *Lactobacillus plantarum* prolonged survival of tumor-bearing mice by enhancing effector Cd8 + T cells functions, Cd4 + T cells differentiation, and NK cells intratumoural infiltration.^119^ In a trial probiotics (*Lactobacillus acidophilus* NCFM and *Bifidobacterium lactis* BI-04) were given to 15 CRC patients (NCT03072641,^120^). Enrichment of butyrate-producing bacteria (e.g. *Clostridiales* and *Faecalibacterium*) in both tumor and nontumor colonic mucosa, and reduction of CRC-associated genera including *Fusobacterium* and *Peptostreptococcus* in the fecal microbiota was identified. These findings reveal the capability of probiotics to relieve dysbiosis and ameliorate anticancer immunity, yet it remains elusive how such alteration in the microbial profile can benefit cancer patients.

Uprising interest in coupling cancer immunotherapy with probiotics has been emerged. In 2019 Zhuo et al. reported that administration of *L. acidophilus* lysates to carcinogen-treated mice partially restored CRC-associated dysbiosis (e.g. enrichment of *Proteobacteria*) and improved anti-CTLA-4 efficacy, which is attributed to decreased intratumoural populations of Cd4+ Cd25+ Foxp3+ T_Reg_, and increased effector Cd8+ and memory T cells.^121^ In other studies, oral gavage of four *Bifidobacterium* species or *Lactobacillus reuteri* could abrogate the onset of blockade-induced colitis via promoting T cell-mediated metabolism^31^ or lowering expression of group 3 innate lymphocytes^122^ respectively. *E. coli* Nissle 1917 supplementation to tumor-bearing mice also enhanced tumor-specific T cell infiltration and dendritic cell activation to relieve the immunosuppressive tumor microenvironment, thereby ameliorating the efficacy of galunisertib (an immunotherapy drug for transforming growth factor-β blockade but previously displayed poor clinical results).^123^ Nevertheless, although probiotics have been widely popularized in the general public, there are conflicting clinical results for many probiotic strains and formulations with inadequate understandings about their impacts on host and interactions with the commensal microbiota.^124^

### Fecal microbiota transplantation

FMT is a therapy to deliver feces from healthy donors into the gastrointestinal tract of receivers via colonoscopy or oral administration to cure diseases by restoring the balance and functions of gut microbiota. FMT has been widely applied to treat recurrent *Clostridium difficile* infection with incredibly high response rates (≥90%),^125,126^ and it also shows its therapeutic potential against graft-versus-host disease, neuropsychiatric (e.g. depression and Parkinson’s disease) or other gut disorders (e.g. IBD and ulcerative colitis).^127,128^ Whereas to date evidence on using FMT to treat gastrointestinal cancer is vastly limited. In the study of Wong et al., the transfer of fecal samples from CRC patients into carcinogen-treated microbiota-depleted mice resulted in increased intestinal carcinogenesis,^129^ yet whether acquiring feces from healthy individuals could suppress CRC progression requires testing. Additionally, restoration of gut microbial diversity (e.g. enrichment of *Lactobacillus* and butyrate-producing taxa) and decrease in intrahepatic lipid accumulation were observed in mice with high-fat-diet-induced steatohepatitis after acquiring feces from healthy mice, suggesting that FMT could mitigate the onset of nonalcoholic fatty liver disease-related HCC.^130^

Similarly, coupling FMT with immunotherapy is mainly under preclinical investigation. Transplanting feces from responders of anti-PD-1 treatment into tumor-bearing mice with depleted microbiota could ameliorate PD-1/PD-L1 blockade efficacy.^26,29,30^ Such improvement is attributed to altered T cell repertoire: enrichment of Cd45+ and effector Cd8 + T cells, and reduction of Cd11b+Cd11c+ MDSCs, RORγt+ T_H_17, and Cd4+ Foxp3+, and Cd4+ Il-17+ T_Reg_, which is in line with clinical findings.^29^ In 2018 FMT was pioneering employed to treat two patients with steroid- and immunosuppressive-refractory ICI blockade-induced colitis (NCT1928394 for the first case and NCT02113657 for the second case^131^). Both patients experienced complete remission upon ≤2 times of FMT with reduced inflammation and increased CD4+ FoxP3+ T_Reg_-to-effector CD8 + T cells ratio in colonic mucosa. Microbial community reconstruction was observed in both patients with a notable enrichment of *Bifidobacterium*, which was previously illustrated its ability to abrogate CTLA-4-blockade-induced colitis in mice^31^. Currently, there is one ongoing early-phase 1 trial to test whether FMT can improve immunotherapy efficacy in anti-PD-1-resistant/refractory gastrointestinal cancer patients (NCT04130763; Table 3). Overall FMT has an excellent safety profile in treating nonmalignant disease even in immunocompromised patients.^132,133^ As for cancer, in 2019 Wardill et al. described the current limitations on utilizing FMT as supportive cancer therapy involving highly varied definition and delivery methods across the globe.^134^ More importantly, it is impossible to standardize the approach due to the difficulty in defining “healthy” microbiota; thus, the risk of disease transmission should never be neglected, and perhaps transplantation of known beneficial microbes or probiotics would be a better alternative to FMT.

## Current limitations and future directions

The popularization of next-generation sequencing has brought a tremendous breakthrough in deciphering the features of human gut microbiota in health and cancer states. Subsequent preclinical investigations have evaluated the mechanistic link between microbes and host immunity in carcinogenesis as exemplified by the discovery of *F. nucleatum* enrichment in metagenomic studies,^135^ following by animal work to illustrate its immunosuppressive feature to promote CRC.^136,137^ Numerous current findings have displayed that microbes can affect anticancer immunity. Yet, these works mostly focuses on elucidating the role of specific species instead of a microbial community in the cancer-immunity crosstalk. To date how the altered microbiota as observed in gastrointestinal cancer patients is correlated with the suppressed immune system is vastly unknown, and in fact this is in line with one of the key limitations in the majority of microbial profiling publications – descriptive findings are mostly provided without in-depth explanation. Indeed, it is now generally accepted that microbiota in cancer patients is distinct from noncancer individuals, but the mechanistic correlation between such compositional difference and cancer progression remains massively unclear. Hence, more efforts are suggested to link sequencing data with the extensive investigation when conducting microbiota research. For example, an ongoing clinical trial aims to determine the association of gut microbiota with neutrophils intratumoral infiltration in CRC (NCT03841799; Table 3).

There are several more unaddressed issues regarding to the study on gut microbiota. For instance, Walter *et al*. in 2020 reported the unrealistic high rate of pathologies being transferred from humans to rodents among publications (95%) and expressed their concerns as these findings may overstate the roles of microbiota in human diseases.^138^ They, therefore, recommended that a more rigorous experimental approach is required worldwide to avoid false concepts. In 2019 the International Cancer Microbiome Consortium (ICMC) released an expert consensus on the role of the microbiome in cancer initiation with the highlight of five pivotal questions (description in Table 4) and suggesting the necessity of conducting large interventional cohort studies with integrative analysis with other oncological research projects to comprehensively reveal the microbe-associated cancer-related pathologies (e.g. genotoxicity, suppressed immunity, and altered metabolism).^139^

**Table 4.** Summary of the consensus statement of the International Cancer Microbiome Consortium.^139.^

Key Aspect	Statement	Suggestion
Relevance of dysbiosis in carcinogenesis	No definition of a “normal” microbiome Microbiome could be pathology-related in a person but not in another person.	Define dysbiosis according to its functional features. Consider dysbiosis as a persistent departure from health-related homeostatic state to cancer-promoting phenotype.
Mechanisms of microbiome-induced carcinogenesis	Five potential mechanisms	Genetic integration, inflammation, and metabolism are supported by human studies. Genotoxicity and immunity are supported mainly by animal work.
Conceptual frameworks describing how microbiome may drive carcinogenesis	Inadequate human evidence to support the renowned “driver-passenger” model.^140^	A new hypothesis is proposed. Carcinogenesis is the outcome of harmful, tripartite, multidirectional interactions among microbiome, environment, and epigenetically or genetically valuable host.
Relationship between microbiome and cancer aetiopathogenesis	Well established that single microbial species can promote carcinogenesis. E.g. *Helicobacter pylori* in gastric cancer	Weak evidence from human studies to show that a microbial community can induce cancer. Direct human evidence is lacking as current studies have been cross-sectional with single time-point sampling.
Future directions	Large, international cohort studies Prospective longitudinal sampling Interventional rather than observational studies Integrative analysis with other oncology research Standardization when presenting microbiome data with enhanced transparency.	

It is now clear that gut microbiota has an unneglectable role in influencing cancer immunotherapy. Such discovery has provided solid fundamentals to ameliorate treatment efficacy by modulating the microbial profile in patients via distinct approaches such as probiotic supplementation and FMT. Yet again, the lack of understanding of the mechanistic crosstalk between host immunity and specific microbes or overall microbial community has limited the progress of the clinical investigation. To increase the translational potential of microbiota-targeted therapies, ICMC pinpointed the importance of formulating standardized guidelines with enhanced transparency to facilitate reproducibility when presenting “meta-omics” data in the academia.^139^ Additionally, combining microbiota research with other novel technologies may offer new findings to connect with scientific areas that have been well studied. For example, the patient-derived organoid model is a newly developed and robust *in vitro* system, which has shown great potential in predicting treatment outcomes due to the complete preservation of phenotypes and genotypes of their originated tumors.^141^ A study in 2020 performed whole-genome sequencing on human CRC organoids with exposure to genotoxic *pks*+ *E. coli* to reveal its related oncogenic mutational signatures, thus illustrating the use of organoids to depict the microbe-associated carcinogenesis at the genomic level.^142^

Furthermore, it has been increasingly documented that metabolites (referring to the intermediate end products during microbe-mediated metabolism) have distinct roles in cancer progression. For example, a class of metabolites known as bile acids has demonstrated its cancer-promoting properties under dysbiotic condition.^143^ Alterations in microbiota composition could increase the level of deoxycholic acid (a type of bile acid known for causing DNA damage^144^) in the enterohepatic circulation, leading to disruption in the gut barrier and further promoting intestinal^145^ or liver carcinogenesis.^146^ Of note, evidence on how metabolites could influence immunotherapy had been insufficient until recently, by which a study in 2020 identified that a metabolite called inosine (produced by *Bifidobacterium pseudolongum*) could enhance response to immune checkpoint blockade in mice through activating anticancer T cells.^147^ In addition to bacteria, accumulating publications have displayed the importance of gut viral, fungal, and archaeal microbiota in gastrointestinal cancers including CRC.^148–150^ Yet, it is noteworthy that to date there is a lack of investigation on their roles in cancer treatment, and hence future work (e.g. shotgun metagenomic sequencing, which allows cross-domain profiling with greater taxonomy resolution and genomic coverage than conventional 16S rRNA sequencing) is suggested to evaluate whether nonbacterial microbiota can also affect the efficacy of immunotherapy.

## Conclusion

Coupling the modern sequencing technology with the mechanistic investigation has aided the identification of novel interaction between the host immunity and microbial community in health and cancer states. Here we highlight the importance of gut commensals on modulating the efficacy of cancer immunotherapy. Notably, strategies that target microbiota as a cancer monotherapy or an adjuvant of first-line treatment have now been heavily studied. In summary, even though the knowledge gap between gut microbiota and gastrointestinal cancer is narrowing, enormous efforts are undoubtedly needed to fully unravel the underlying mechanisms, thereby potentiating the application of microbiota-targeted therapies in clinical practice.

## Supplementary Material

Supplemental Material

## Figures and Tables

**Table:** 

ACT	Adoptive cell transfer
CAR	Chimeric antigen receptor
CD	Cluster of differentiation
CRC	Colorectal cancer
CTLs	Cytotoxic T lymphocytes
CTLA-4	Cytotoxic T lymphocyte-associated antigen-4
dMMR	DNA mismatch repair-deficient
FMT	Faecal microbiota transplantation
HCC	Hepatocellular carcinoma
ICIs	Immune checkpoint inhibitors
ICMC	International Cancer Microbiome Consortium
IgA	Immunoglobulin A
IL	Interleukin
irAEs	Immune-related adverse events
LPS	Lipopolysaccharides
MAMPs	Microbe-associated molecular patterns
MDSCs	Myeloid-derived suppressor cells
MSI-H	Microsatellite instability-high
NK	Nature killer
NSCLC	Nonsmall-cell lung cancer
ODNs	Oligodeoxynucleotides
PFS	Progression-free survival
PD-1	Programmed cell death protein-1
PD-L1	PD-1 ligand 1
RCC	Renal cell carcinoma
TBI	Total body irradiation
TCR	T cell receptor
T_H_	T helper cells
TILs	Tumour-infiltrating lymphocytes
TIM	Tumour-immune microenvironment
TLR	Toll-like receptor
TNF	Tumour necrosis factor
