# Supplementary material for: Gut microbiota: impacts on gastrointestinal cancer immunotherapy
Source: Gut Microbes. 2021 Jan 12;13(1):1869504. doi: 10.1080/19490976.2020.1869504 (PMC7808428; doi:10.1080/19490976.2020.1869504)
Supplement: Supplemental Material [file KGMI_A_1869504_SM9425.docx]

**Table 1. Summary on the association of gut microbiota with host immunity to impact cancer immunotherapy.**

| Immuno-therapy | Influence on Therapy | Phylum | Bacterium | Host | Correlation with Host Immunity | Ref. |
| --- | --- | --- | --- | --- | --- | --- |
| ACT | Enhancement | *Bacteroidetes* | *Bacteroides,*  *Parabacteroides* | TB Mice | Raise systemic Cd8α+ DCs; Induce T_H_1-mediated immune response | [41] |
|  |  | - | Gram-negative LPS-producing bacteria | TB Mice | Induce microbial translocation into lymph nodes to activate Tlr4 signalling for PIC production | [40] |
|  | Regression | *Bacteroidetes* | *S24-7* | TB Mice | - | [41] |
| Anti-CTLA-4 Therapy | Enhancement | *Bacteroidetes* | *Bacteroides fragilis* | TB Mice | Induce T_H_1-mediated immune response and DC maturation in tumour-draining lymph nodes | [61] |
|  |  | *Firmicutes* | *Faecalibacterium, Lachnospiraceae, Ruminococcus* | Melanoma Patients | Relate to low systemic T_Reg_, CD4+ and CD8+ T cells at baseline; Raise systemic CD4+ T cells upon treatment initiation | [79] |
|  |  | *Proteobacteria* | *Burkholderia cepacia* | TB Mice | Induce T_H_1-mediated immune response and DC maturation in tumour-draining lymph nodes | [61] |
| Anti- PD-1/ PD-L1 Therapy | Enhancement | *Actinobacteria* | *Bifidobacterium abreve* | TB Mice | Induce DC-mediated immune response, T cell activation and PIC production | [63] |
|  |  |  | *Bifidobacterium adolescentis* | TB Mice | Induce DC-mediated immune response, T cell activation and PIC production | [63] |
|  |  |  |  | Melanoma R | Raise tumour-specific CD8+ T cells | [65] |
|  |  |  | *Bifidobacterium dentium* | HCC R | Relate to anti-inflammation | [68] |
|  |  |  | *Bifidobacterium longum* | TB Mice | Induce DC-mediated immune response, T cell activation and PIC production | [63] |
|  |  |  |  | Melanoma R | Raise tumour-specific CD8+ T cells | [65] |
|  |  |  |  | NSCLC & RCC R | Raise systemic memory CD8+ T cells & NK cells | [67] |
|  |  |  | *Enterococcus faecium* | Melanoma R | Raise tumour-specific CD8+ T cells | [65] |
|  |  | *Bacteroidetes* | *Alistipes putredinis,*  *Prevotella corpri* | NSCLC & RCC R | Raise systemic memory CD8+ T cells & NK cells | [67] |
|  |  |  | *Parabacteroides merdae* | Melanoma R | Raise tumour-specific CD8+ T cells | [65] |
|  |  | *Firmicutes* | *Faecalibacterium* | Melanoma R | Raise systemic and intratumoural effector CD4+ and CD8+ T cells | [64] |
|  |  |  | *Lactobacillus* | Melanoma R | Raise tumour-specific CD8+ T cells | [65] |
|  |  |  |  | HCC R | Inhibit inflammatory response by reducing oxidative stress injury | [68] |
|  |  |  | *Ruminococcus obeum***^a^** | HCC R | Inhibit inflammatory response by reducing oxidative stress injury | [68] |
|  |  |  | *Veillonella parvula* | Melanoma R | Raise tumour-specific CD8+ T cells | [65] |
|  |  | *Proteobacteria* | *Klebsiella pneumoniae***^b^** | Melanoma R | Raise tumour-specific CD8+ T cells | [65] |
|  |  | *Verrucomicrobia* | *Akkermansia muciniphila* | NSCLC & RCC R | Induce IL-12-dependent intratumoural infiltration of Ccr-9+Cxcr-3+Cd4+ T cells | [66] |
|  |  |  |  | HCC R | Relate to anti-inflammation | [68] |
|  | Regression | *Bacteroidetes* | *-* | Melanoma R | Raise systemic T_Reg_ & MDSCs | [64] |
|  |  |  | *Bacteroides* | Melanoma Patients | Relate to high systemic CD4+ T cells at baseline; No increase in T cell induction upon treatment initiation | [79] |
|  |  | *Firmicutes* | *Ruminococcus (obeum***^a^***)* | Melanoma, RCC & NSCLC NR | - | [65, 67] |
|  |  | *Proteobacteria* | *Escherichia coli, Klebsiella pneumoniae***^b^** | HCC NR | - | [68] |
| ICI-Induced irAEs | Regression | *Actinobacteria* | *Bifidobacterium* | DSS-TB Mice | Induce T cell-related metabolism | [62] |
|  |  | *Bacteroidetes* | Bacteroidaceae, Barnesiellaceae, Rikenellaceae | Colitis-Free Melanoma Patients | Induce polyamine transport and B vitamin biosynthesis | [76] |
|  |  |  | *Bacteroides uniformis*, *Parabacteroides distasonis, Prevotella* | Melanoma Patients | Relate to high systemic CD4+ T cells at baseline; No increase in T cell induction upon treatment initiation | [79] |
|  | Enhancement | *Firmicutes* | *Clostridiales bacterium, Faecalibacterium prausnitzii* | Melanoma Patients | Relate to low systemic T_Reg_, CD4+ and CD8+ T cells at baseline; Raise systemic CD4+ T cells upon treatment initiation | [79] |
| CpG-ODN Therapy | Enhancement | *Bacteroidetes* | *Alistipes shahii* | TB Mice | Induce TNF production by tumour-infiltrating lymphocytes and tumour-associated myeloid cells | [90] |
|  |  | *Firmicutes* | *Ruminococcus* |  |  |  |
|  |  | - | Gram-negative LPS-producing bacteria |  |  |  |
|  | Regression | *Firmicutes* | *Lactobacillus* | TB Mice | - | [90] |

**^a^** Contradictory findings as being correlated with both enhancement and regression of the efficacy of anti-PD-1/PD-L1 therapy.

**^b^** Contradictory findings as being correlated with both enhancement and regression of the efficacy of anti-PD-1/PD-L1 therapy.

*Abbreviations:* DC, Dendritic cells; NR, Non-responders; PIC, Pro-inflammatory cytokines; R, Responders; TB, Tumour-bearing.

**Table 2. FDA-approved immune checkpoint blockade against gastrointestinal cancer.**

| Target | Drug Name | Brand Name | Indication for GIC^a^ | Reference |
| --- | --- | --- | --- | --- |
| PD-1 | Nivolumab | Opdivo | HCC  dMMR/MSI-H CRC | NCT01658878 [44] NCT02060188 [49] |
|  | Pembrolizumab | Keytruda | GC  ESCC  HCC  dMMR/MSI-H CRC | NCT02335411 [46]; NCT02370498 [47] NCT03189719 [48] NCT02702414 [45]  NCT02054806 [50] |
|  | Cemiplimab | Libtayo | - | - |
| PD-L1 | Atezolizumab | Tecentriq | - | - |
|  | Avelumab | Bavencio | - | - |
|  | Durvalumab | Imfinzi | - | - |
| CTLA-4 | Ipilimumab | Yervoy | - | - |
| Combined | Nivolumab plus Ipilimumab | Opdivo & Yervoy | dMMR/MSI-H CRC | NCT02060188 [49, 58] |

^a^ Unless further specification, all included indications are applied as monotherapy.

*Abbreviations:* ESCC, Oesophageal squamous cell carcinoma; GC, Gastric cancer.

**Table 3. Ongoing clinical studies on the role of microbiota in gastrointestinal cancer.^a^**

| Study Type | Trial Information | Study Model | Time Perspective | Patient Population | Intervention | Study Aim |
| --- | --- | --- | --- | --- | --- | --- |
| Observational | NCT01313442; US; R | Cohort | Prospective | GIC (*n* = 500) | - | Cohort establishment |
|  | NCT02726243; France; R | Case-Control | Prospective | Healthy, IBD, CRC (*n* = 240) |  |  |
|  | NCT03998644; China; R | Case-Control | Cross-Sectional | Healthy, CRA, CRC (*n* = 2000) |  |  |
|  | NCT04015466; EU & CELAC; R | Cohort | Prospective | GC (*n* = 800) |  |  |
|  | NCT04189393; Netherlands; NR | Cohort | Prospective | GIC (*n* = 60) |  | Map the oral and gut microbiome in patients |
|  | NCT03623152; Hong Kong; R | Case-Only | Cross-Sectional | Healthy, CRA, CRC (*n* = 160) |  | Compare microbiota in left and right colon |
|  | NCT03841799; France; R | Cohort | Prospective | CRC (*n* = 80) |  | Study gut microbiota and immune infiltration |
|  | NCT03385213; China; R | Case-Control | Retrospective | Patients with relapse CRC (*n* = 200) |  | Study gut microbiota in patients with relapse CRC |
|  | NCT03667495; China; R | Other | Prospective | Patients with relapse CRC (*n* = 100) |  | Study gut and oral microbiota in patients with relapse CRC |
|  | NCT04005118; France; R | Cohort | Prospective | CRC (*n* = 50) |  | Study association of gut microbiota with post-operative complications |
|  | NCT04071964; Canada; R | Other | Prospective | CRC (*n* = 300) |  | Study association of gut microbiota with healing after surgery |
|  | NCT03191110; Netherlands; R | Cohort | Prospective | CRC (*n* = 2000) |  | Study association of lifestyle factors with CRC survival and recurrence |
| Interventional; Early Phase 1 | NCT04130763; China; R | Single Group Assignment | - | Anti-PD-1 resistant or refractory GIC (*n* = 5) | 2-week oral FMT from donors with similar microbiota as in anti-PD-1 responders | Test whether FMT can improve efficacy in anti-PD-1 resistant or refractory patients |
| Interventional; Phase 2 | NCT03359681; Denmark; R | Parallel Assignment |  | CRC (*n* = 48) | Metformin HCL 20 days before and 10 days after surgery or placebo | Test the drug effects on tumour cell growth, immunological and metabolic change in CRC patients |
|  | NCT03661047; US; R | Parallel Assignment |  | CRC (*n* = 36) | 2-year daily intake of marine omega-3 fatty acid (4-gram) with treatment of AMR101 or placebo | Test the drug effects on TIM and microbiome; Examine the drug effects on tumour pathologic and molecular features prior to any other therapies |
|  | NCT03831698; US; R | Singe Group Assignment |  | CRC, Lynch Syndrome (*n* = 34) | 12-month daily intake of omega-3 fatty acid ethyl esters (2-gram) | Test the drug effects on molecular and microbiota changes in patients |
|  | NCT03781778; US; R | Parallel Assignment |  | Stage I to III CRC survivors (*n* = 24) | 8-week intake of resistant starch or regular corn starch as control | Test the dietary effects on inflammation, insulin resistance and microbiota in survivors |
| Interventional; Not Applicable | NCT03028831; US; R | Single Group Assignment |  | Healthy, CRA (*n* = 60) | 4-week intake of resistant starch or digestible starch as control | Test the dietary effects on lowering CRC risk, adenoma recurrence and inflammation |

**^a^** Clinical studies included were searched from <clinicaltrials.gov> using the following 2 key words: “gastrointestinal cancer” and “microbiota”. Completed trials were excluded, as well as investigations on the relationships between microbiota and chemotherapy/radiotherapy due to their irrelevancy to this article. The search was conducted in April 2020.

*Abbreviations:* CRA, Colorectal adenoma; GIC, Gastrointestinal cancer; HCL, Hydrochloride; NR, Not yet recruiting; R, Recruiting.

**Table 4. Summary on the consensus statement of the International Cancer Microbiome Consortium [139].**

| Key Aspect | Statement | Suggestion |
| --- | --- | --- |
| Relevance of dysbiosis in carcinogenesis | - No definition of a “normal” microbiome - Microbiome could be pathology-related in a person but not in another person | - Define dysbiosis according to its functional features - Consider dysbiosis as a persistent departure from health-related homeostatic state to cancer-promoting phenotype |
| Mechanisms of microbiome-induced carcinogenesis | - 5 potential mechanisms | - Genetic integration, inflammation and metabolism are supported by human studies - Genotoxicity and immunity are supported mainly by animal work |
| Conceptual frameworks describing how microbiome may drive carcinogenesis | - Inadequate human evidence to support the renowned “driver-passenger” model [150] | - A new hypothesis is proposed - Carcinogenesis is the outcome of harmful, tripartite, multidirectional interactions among microbiome, environment and epigenetically or genetically valuable host |
| Relationship between microbiome and cancer aetiopathogenesis | - Well-established that single microbial species can promote carcinogenesis - E.g. *Helicobacter pylori* in gastric cancer | - Weak evidence from human studies to show that a microbial community can induce cancer - Direct human evidence is lacking as current studies have been cross-sectional with single time-point sampling |
| Future directions | - Large, international cohort studies - Prospective longitudinal sampling - Interventional rather than observational studies - Integrative analysis with other oncology research - Standardisation when presenting microbiome data with enhanced transparency | |
